# Supplementary material for: Self-supervised maize kernel classification and segmentation for embryo identification
Source: Front Plant Sci. 2023 Apr 14;14:1108355. doi: 10.3389/fpls.2023.1108355 (PMC10140504; doi:10.3389/fpls.2023.1108355)
Supplement: Supplementary file 1 [file DataSheet_1.docx]

**Self-Supervised Maize Kernel Classification and Segmentation for Embryo Identification**

David Dong^1,2^, Koushik Nagasubramanian^2,3^, Ruidong Wang^4^, Ursula K Frei^4^, Talukder Z Jubery*^2,5^, Thomas Lübberstedt^4^, Baskar Ganapathysubramanian*^2,5^

^1^Ames High School, Ames, IA, USA

^2^Translational AI Center, Iowa State University, Ames, IA, USA

^3^Department of Electrical Engineering, Iowa State University, Ames, IA, USA

^4^Department of Agronomy, Iowa State University, Ames, IA, USA

^5^Department of Mechanical Engineering, Iowa State University, Ames, IA, USA

*Corresponding author(s): T Z Jubery, B. Ganapathysubramanian; email: [znjubery@iastate.edu](mailto:znjubery@iastate.edu),

[baskarg@iastate.edu](mailto:baskarg@iastate.edu)

**SUPPLEMENTARY INFORMATION**

**Table S1** Average validation metrics on U-Net from SimCLR, NNCLR and ImageNet pretrained weights

| Learning rate | | 1e-03 | | | | 1e-04 | | | | | | 1e-05 | | | | |
| --- | --- | --- | --- | --- | --- | --- | --- | --- | --- | --- | --- | --- | --- | --- | --- | --- |
| Channel count | | 3 | 4 | 5 | | 3 | 4 | | | 5 | | 3 | | 4 | | 5 |
|  | | SimCLR | | | | | | | | | | | | | | |
| BCE Loss | | 0.132 | 0.163 | 0.206 | | 0.135 | 0.171 | | | 0.213 | | 0.099 | | 0.116 | | 0.155 |
| Accuracy | | 0.963 | 0.965 | 0.964 | | 0.962 | 0.967 | | | 0.966 | | 0.961 | | 0.963 | | 0.963 |
| Dice Score | | 0.797 | 0.807 | 0.810 | | 0.761 | 0.828 | | | 0.821 | | 0.764 | | 0.797 | | 0.771 |
|  | | NNCLR | | | | | | | | | | | | | | |
| BCE Loss | | 0.131 | 0.164 | 0.163 | 0.163 | | | 0.216 | 0.181 | | 0.100 | | 0.128 | | 0.173 | |
| Accuracy | | 0.950 | 0.954 | 0.972 | 0.961 | | | 0.967 | 0.966 | | 0.954 | | 0.971 | | 0.964 | |
| Dice Score | | 0.792 | 0.791 | 0.794 | 0.793 | | | 0.824 | 0.820 | | 0.723 | | 0.800 | | 0.802 | |
|  | ImageNet | | | | | | | | | | | | | | | |
| BCE Loss | | 0.194 | 0.200 | 0.202 | 0.200 | | | 0.208 | 0.183 | | 0.103 | | 0.196 | | 0.182 | |
| Accuracy | | 0.965 | 0.967 | 0.966 | 0.959 | | | 0.965 | 0.966 | | 0.960 | | 0.964 | | 0.956 | |
| Dice Score | | 0.810 | 0.824 | 0.826 | 0.783 | | | 0.819 | 0.798 | | 0.765 | | 0.676 | | 0.771 | |

**Table S2** Hyperparameter values for best segmentation models for each weight initialization type as found by cross-validation

|  | Filters | Learning rate |
| --- | --- | --- |
| SimCLR | 4 | 1e-04 |
| NNCLR | 4 | 1e-04 |
| ImageNet | 5 | 1e-03 |


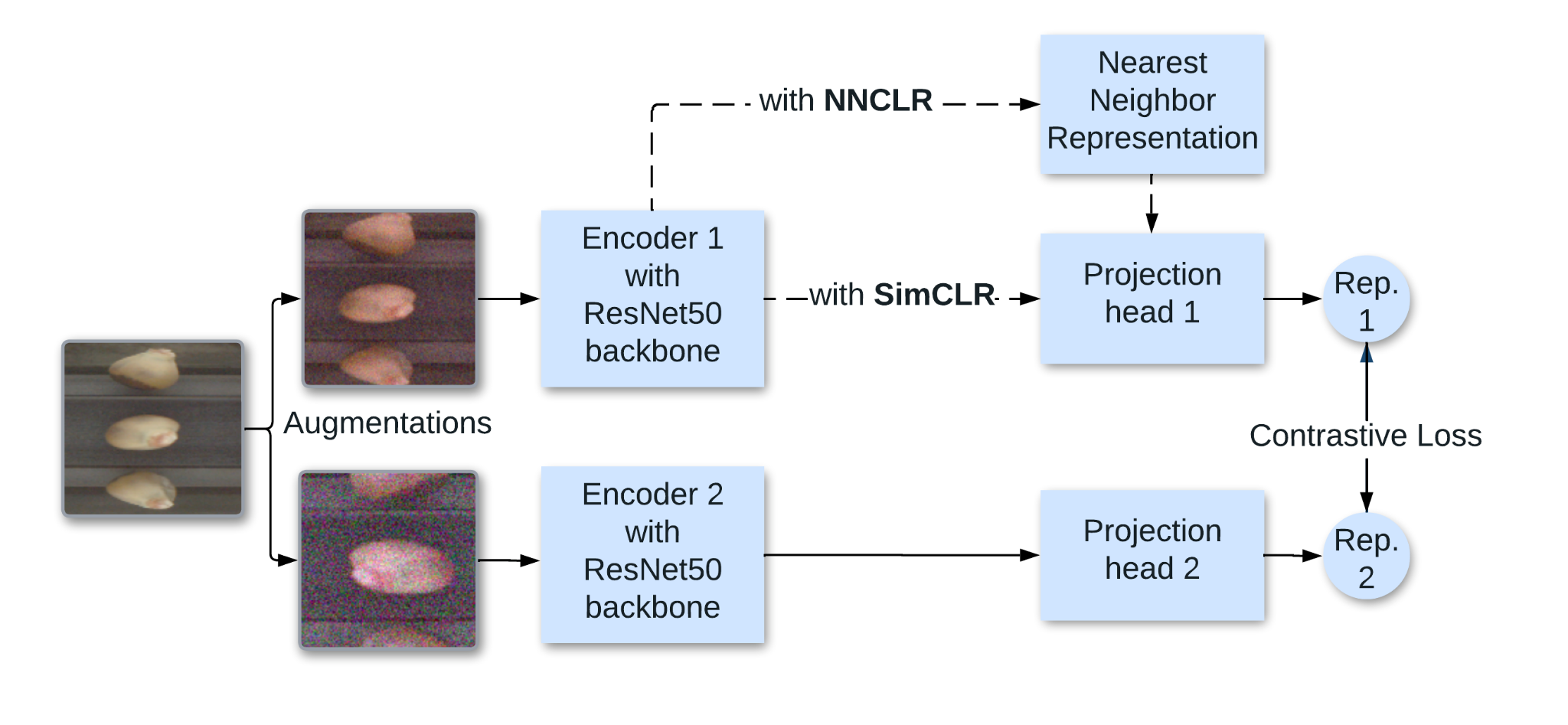


**Figure S1** Superimposed diagram of NNCLR and SimCLR models. The two dashed arrows show the paths a tensor representation passes depending on the self-supervised model.


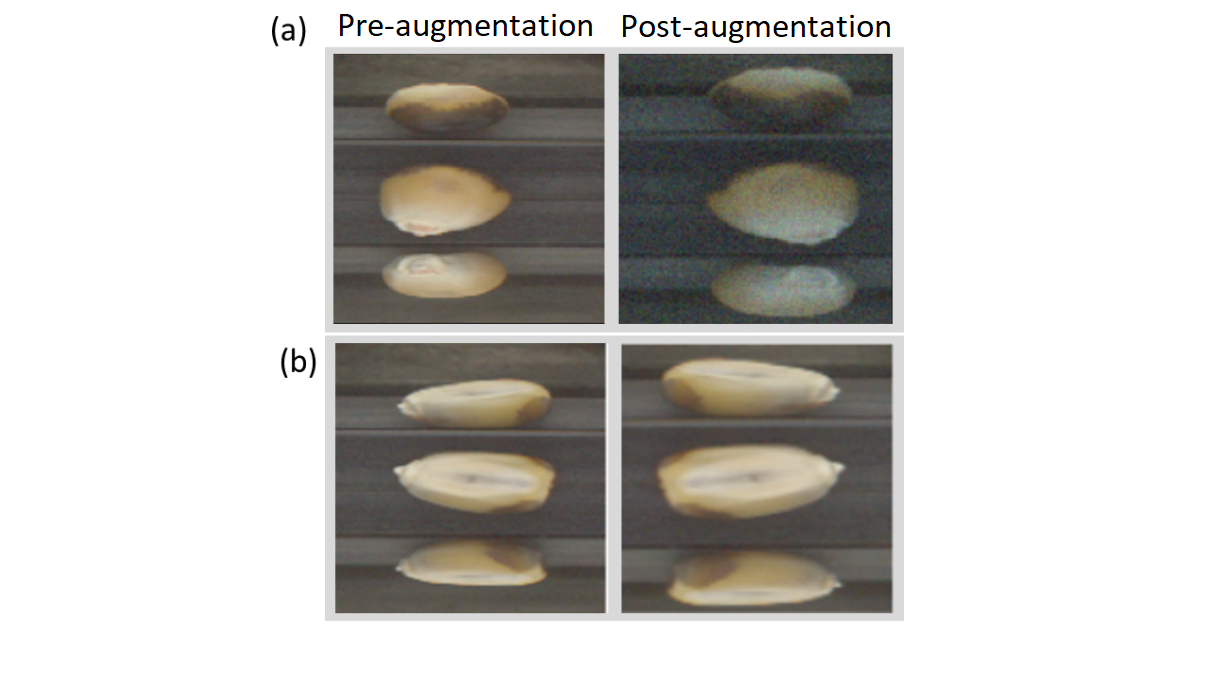


**Figure S2:** Two examples of a processed dataset image and its augmentation. (a) Random zoom, horizontal flip, brightness and color transform, and Gaussian noise. (b) Random zoom and horizontal flip.


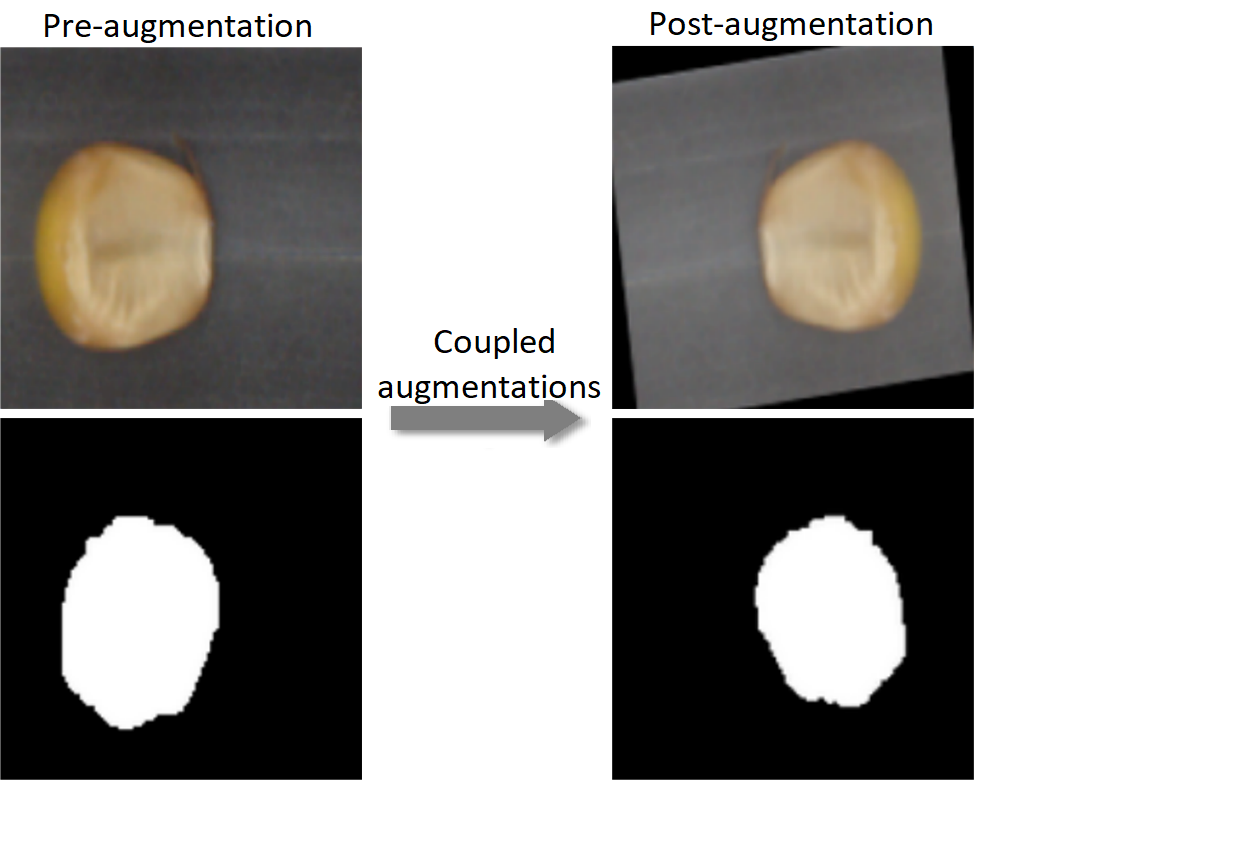


**Figure S3** Coupled augmentations for an RGB image and its mask.


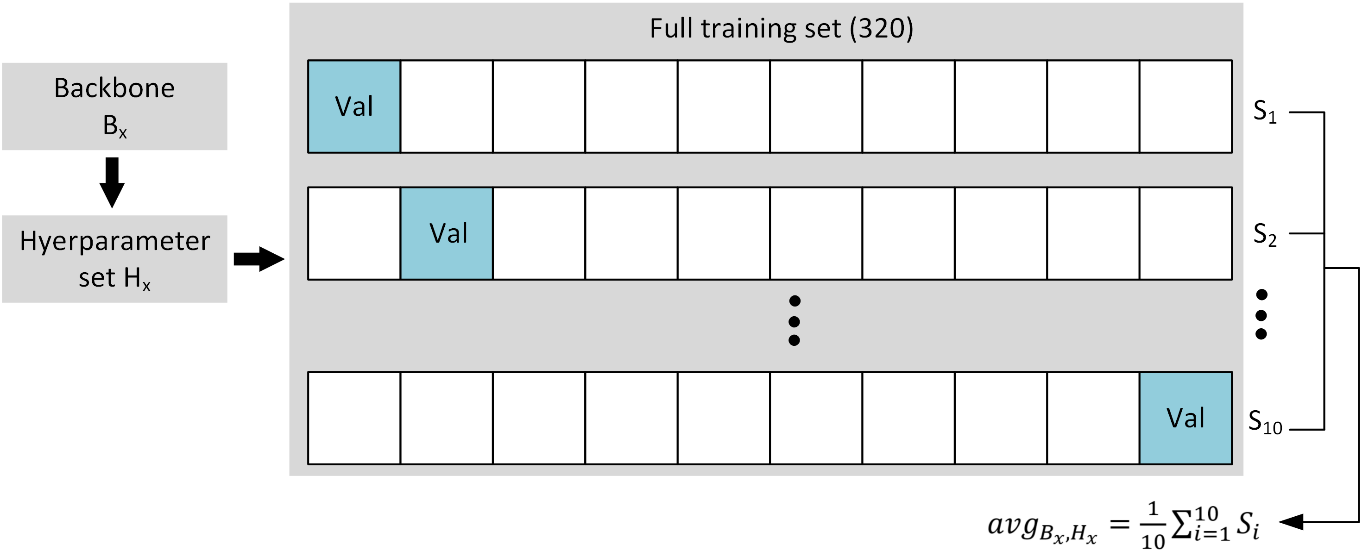


**Figure S4** Cross-validation model selection procedure.
